# A cross sectional survey assessing knowledge, attitudes and behaviors regarding brucellosis among Arab Israelis

**DOI:** 10.1186/s12889-018-5430-9

**Published:** 2018-04-18

**Authors:** Orna Baron-Epel, Shiran Bord, Michal Cohen-Dar, Samira Obeid

**Affiliations:** 1 School of Public Health, Faculty of Social Welfare and Health Studies, University of Haifa, 31905 Mount Carmel, Israel; 2 North District Health Office, Ministry of Health, Nazereth, Israel; 3 Nursing Faculty, The Max Stern Yezreel Valley College, 19300 Yezreel Valley, Israel; 4 Health management Department, The Max Stern Yezreel Valley College, 19300 Yezreel Valley, Israel

**Keywords:** Brucellosis, Consumption of non-pasteurized dairy products, Attitudes, Knowledge, Arabs

## Abstract

**Background:**

Brucellosis is a contagious zoonotic disease transferred from sick animals to humans and endemic in the Middle East and other countries. Humans mainly acquire the disease by consuming non-pasteurized dairy products from infected animals. This study assesses the rates of non-pasteurized dairy product consumption, knowledge and attitudes regarding brucellosis among Israeli Arabs, in towns with and without reported cases of brucellosis. The aim is to assess if there is an association between knowledge, attitudes and consumption of non-pasteurized dairy products and if encountering the disease in the community is associated with consumption, attitudes and knowledge.

**Methods:**

A cross sectional telephone survey of 306 respondents from five Arab towns in the northern part of Israel, three towns with and two without reported cases of the disease during 2014. The questionnaire included questions regarding knowledge and attitudes related to brucellosis and patterns of production, purchase and consumption of dairy products from non-regulated sources, mainly semi-hard low value white cheese.

**Results:**

Nearly 41% of respondents reported consuming cheese from non-regulated sources and 16.1% of respondents reported purchasing milk from non-regulated sources. Favorable attitudes towards factors enhancing transmission of brucellosis were associated with purchasing and consuming milk or homemade white cheese from non-regulated sources in multivariable logistic regression models (odds ratio- 2.21 and 2.66 respectively, confidence intervals between 1.7 and 3.9). However, knowledge about the disease was not associated with these behaviors. In towns with previous reported cases of the disease the purchasing and consumption of non-regulated cheeses was higher than in towns without reported cases and the opposite for non-regulated milk consumption.

**Conclusions:**

The purchase and consumption of cheese from non-regulated sources is very common in specific communities among Israeli Arabs. Attitudes are a significant factor associated with the risky behavior, such as consuming milk and cheese from non-regulated sources. However, knowledge and previous reported cases of the disease in the community do not prevent most risky behaviors. Interventions should not focus only on dissemination of information.

## Background

Most milk and dairy products are made at large dairies that pasteurize the milk before reaching the consumer and are regulated by the authorities. However, in certain communities this is not the only way people attain milk and milk products. It is not clear to what extent milk, and its products, from non-regulated dairies and individual farmers are purchased and consumed in specific communities. It is also not clear to what extent these non-authorized dairies and individual farmers pasteurize their milk. Therefore, the milk may contain numerous pathogens that can contaminate milk products and cause illness and even death [1]. Brucellosis is such a disease and can cause a range of signs and symptoms, some of which may present for prolonged periods of time [2].

Brucellosis is endemic in Israel as in other countries in the Middle East [3–5], South Eastern European countries [6], China [7] and elsewhere and patterns are changing [7, 8]. Studies from Egypt suggest widespread high-risk practices are common and lack of knowledge regarding disease transmission may enhance infection [3, 9]. For example, in Egypt it was reported that although livestock owners have good knowledge regarding brucellosis, they still practiced high-risk behaviors such as not wearing gloves when handling ruminants or selling the animal when it is suspected of having the disease. Many reported making cheese from non-pasteurized milk [3]. In another study in Egypt the authors report lack of knowledge regarding disease transmission, and widespread high-risk practices regarding handling the livestock [9].

In the USA, during 1993–2006, 1571 cases and 2 deaths involving non-pasteurized dairy products were identified. States that restricted sales of non-pasteurized milk products had fewer outbreaks and illness [10], but consumption of non-pasteurized milk is still common mostly in farm families and seems to have increased in recent years [1].

In Israel, *Brucella melitensis* may be present in sheep, goats and sometimes in cattle and camels [11–13]. Since the mid-1980s *Brucella melitensis* has been the only agent of Brucellosis identified in Israel and the majority of human brucellosis cases are caused by ingestion of unpasteurized dairy products from infected animals [14]. During the 80’s and 90′ there was a resurgence of the disease with rates between 1 and 4 cases per 100,000. During this period three outbreaks (over 5 cases per 100,000) occurred, the highest in 1988 with 11 cases per 100,000 [15]. If calculated only within the Arab community the rates reached as high as 60 cases per 100,000 among Arabs in 1988. Most of the cases reported were in the Arab population. In the southern part of Israel most cases are among the Bedouin community [16]. The reported cases may be an under reported rate [16]. Following the high rates of the disease up to the late 80’s interventions were implemented and the rates declined. However, in 1997 the campaign to control brucellosis was terminated [17], local control measures have continued. During the last 15 years the rates of the disease were between 2.5–0.9 cases per 100,000 and there were no major outbreaks [15]. However, the rates began to increase again around 2007 [14]. When the source of exposure was known, 66% were caused by ingesting contaminated food products and 34% from exposure to infected farm animals [14]. In 2014, 600 cases were reported in Israel, a five time increase from 2011 [18]. In a report of 41 cases during a 6 month period in the North of Israel in 2014, these cases were confirmed to have *Brucella melitensis* [11]. This increase in number of cases was confined to a specific village and it is assumed that the number of cases was higher than reported. All of the cases reported purchasing goat cheese from a certain family’s herd of goats [11]. The disease is generally concentrated in a number of towns and villages and not evenly distributed [18].

The increase in number of cases seem to be due to ongoing consumption of non-pasteurized dairy products, mainly milk and white cheese, purchased from non-regulated dairies or individual farmers and homemade. The cheese is a low value semi-hard white cheese found throughout the Middle East, this cheese is also used as an ingredient in many sweet and savory pastries.

In Israel, the Ministry of Health (MOH) performs surveillance and educational efforts to decrease levels of the disease. Brucellosis is one of the diseases that are reported to the MOH by law. Efforts are made to educate and inform the community on the need to pasteurize the milk. These are usually local efforts in response to specific identification of cases. In addition, the Ministry of Agriculture works with the farmers to control and eradicate the disease by vaccinating the animals and diagnosing them when there is suspicion of the disease; both ministries collaborate as much as possible. These interventions have been in place for at least 30–40 years. Due to the increase in the rates of the disease, new interventions need to be developed. More information regarding the knowledge, attitudes and risk behaviors of the population are needed in order to develop these interventions.

The current study aims to assess knowledge and attitudes regarding brucellosis and rates of non-regulated dairy (homemade) products purchased and consumed among Israeli Arabs in towns with and without reported cases of brucellosis, this may help in assessing if exposure to past cases of brucellosis in the individuals’ community affect these behaviors. This will help planning effective public health interventions.

## Methods

### Study design

A cross-sectional study including a telephone random digit dial survey from five towns in the Northern part of Israel. The study was approved by the Ethics Committee of the University of Haifa in Israel (number 051/16).

### Sample and procedure

The study population included adults (18 and older) living in five Arab towns in the northern part of Israel. Towns with and without reported cases were identified. The three towns with the most reported cases during 2014 were chosen (about 100 cases reported in these three town during 2014) and another two towns in which no cases of brucellosis were reported at that time. We matched the towns with no reported cases according to socioeconomic status and ethnic background. Two of the towns are Muslim towns (mostly Muslim people living there), one with reported cases of brucellosis and one without, and the other three towns are Druze towns, two with reported cases and one without.

A random telephone survey of homes from each town was performed using the national list of land phone lines for each town. Random telephone calls to 556 people were made during April to June 2016, 250 refused to answer or stopped the interview in the middle. All together 306 residents were interviewed, about 60 from each town, with a response rate of 55%, 123 interviews with residents from towns with no reported cases of brucellosis and 183 interviews from the towns with reported cases of the disease.

### Measurements

The questionnaire was developed based on the literature and 11 qualitative interviews with individuals from towns with reported cases of brucellosis. Then the questionnaire was pretested before beginning the survey. Attitudes, beliefs and behaviors related to consumption of dairy products expressed in the qualitative interviews served as the basis for the items measuring attitudes and behaviors.

Socio-demographic characteristics included gender, age, education (number of years spent at school and higher education), marital status (single or living with spouse), and income (“the average total income of an average household is 18,000 New Israeli Shekels, is your income above (3), average (2) or below the average (1) income”). The interviewees also reported in which town they live and two categories were formed: towns with reported cases (1) and towns with no cases reported (0).

Knowledge was measured with five items stating a fact regarding the transmission of the disease, the disease itself, treatment and prevention (Table 1). The respondent could choose between the answers: correct, incorrect and do not know. “Correct” was scored 1 and “incorrect” or “do not know” were scored 0. The sum of the five items is the score for the knowledge variable, ranging from 0 to 5.

**Table 1:** Response to items depicting knowledge regarding transmission of brucellosis. Percent and Number

		Correct answers % (N)		
		Type of town with or without reported cases		
	Knowledge Items	With	Without	Total
1	The Brucella bacteria can pass from a sick animal to the milk*	73.3 (132)	59.0 (72)	67.5 (204)
2	The Brucella bacteria can cause a serious disease and even death*	85.8 (157)	71.9 (87)	80.3 (244)
3	The disease caused by Brucella causes weakness and high temperature*	87.4 (160)	67.8 (82)	79.6 (242)
4	The disease can be treated in humans with medication	78.1 (143)	71.3 (87)	75.4 (230)
5	All the bacteria can be killed when the milk is pasteurized or boiled to at least 63 degrees (C^0^)	64.5 (118)	59.8 (73)	62.6 (191)
	Mean knowledge* (Range- 0- no correct answers, 5 all correct answers range of 0–5)	3.88	3.29	3.6

* *p* < 0.05 chi square and ANOVA test

Attitudes towards factors that enhance transmission of the disease were measured by seven items (Table 2) rated on a scale of 1–5 where 1 represented “do not agree” and 5 represented “agree very much”. The mean of the items was calculated for each respondent. The items were based on attitudes expressed by the interviewees in the qualitative interviews including two items on fatalism. The measure of internal consistency, Cronbach’s alpha, was 0.715 and in a factor analysis all items loaded onto the same component. The higher the score of the attitudes scale, the more favorable attitudes were towards factors enhancing transmission of the disease.

**Table 2:** Attitudes towards factors enhancing transmission of brucellosis^a^

	Item	Mean	SD	% agree with item (scores 5–4)
1	In my opinion cheeses made at home are tastier than chesses bought in sealed packaging certified by the Ministry of Health	3.03	1.49	29.5
2	I think that milk products from well known dairies are not so fresh as those made at home	3.36	1.56	35.3
3	I am sure that the home made cheeses that I buy are not infected with bacteria as I buy them from people that keep everything clean	2.70	1.48	44.1
4	I have full trust in the people I buy the cheese from	2.95	1.51	38.2
5	I think that if I heat the milk to at least 63 degrees (C^0^) I will kill the Brucella bacteria	3.38	1.42	24.8
6	I think everything is from God, if I need to get sick I will get sick, this has nothing to do with what I eat or drink	2.95	1.73	45.8
7	Getting sick is a matter of luck, If I need to get sick I will get sick, and it has nothing to do with what I eat or drink	3.12	1.67	41.5
	Total scale (mean of 7 items)	3.17	1.01	–

^a^ On a scale of 1–5, 1 represents- do not agree at all, and 5 represents- agree very much

Consumption of milk and white cheese was measured by 13 yes/no questions, including place of purchase, consumption, and preparation of white cheese. All questions referred to milk and cheese from non-regulated farms, as people did not know if the farmers or individuals making the cheese at home had pasteurized the milk, this was obvious from the qualitative interviews. We assume that at least some of these farms do not pasteurize their milk before making the cheese. Four of the questions were chosen for further analysis as they were the most frequent risk practices reported (see Table 4).

### Statistical analysis

Statistical analysis included Chi square tests (for knowledge, behavior and type of town), T test (for attitudes and type of town, and behaviors and attitudes), Mann-Whitney (for behaviors and knowledge) and ANOVA (for knowledge and type of town) tests. We run multivariable logistic regression models, presenting odds ratios, confidence intervals and *p* values. The variables added to the regression model were those associated with the dependent variables (the behaviors) and having low multicollinearity with other variables in the model. Socioeconomic variables were adjusted for. An enter method was used when running the models. Goodness of fit was assessed using the Hosmer and Lemeshow statistic and the Nagelkerke R^2^.

## Results

The study population included 306 respondents from five towns, in the northern part of Israel, the mean age was 43.6 ± 15.6 and 35.3% were men. Seventy percent of the respondents were married and 70% had children, 28% had not finished high school, 68% reported having below average income. These results correspond to the Arab populations’ characteristics in Israel.

Table 1 describes the level of knowledge regarding the disease and its transmission and prevention by type of town (with or without previous reported cases). Generally, between 60% and 80% of respondents knew the correct answer. Knowledge was significantly higher in towns with previous reported cases of the disease.

Older respondents reported significantly better knowledge regarding transmission of the disease (r_s_ = 0.25) and women reported higher levels of knowledge (*p* = 0.05). Education was not associated with knowledge, but those with lower income actually had higher levels of knowledge (*p* = 0.038) (data not presented).

Table 2 presents the seven items that assessed the attitudes of the respondents towards factors enhancing transmission of the disease (Cronbach’s alpha 0.715). A large proportion of respondents agreed with the items depicting fatalistic attitudes (41–46%, the two last items in Table 2). About 40% of respondents trusted the people from whom they bought the homemade cheese. Nearly 30% agreed with the item referring to the better taste of homemade cheese. The sum of the attitudes towards factors that enhance transmission of the disease were not associated with age and gender, but were significantly associated with education (*p* < 0.0001) and income (*p* = 0.029). Those with less education and lower income had higher levels of favorable attitudes towards factors enhancing transmission. There was no significant difference in levels of attitudes between the two types of towns, (3.12 ± 0.98 compared to 3.04 ± 0.944 respectively *p* = 0.08).

Over 90% of the respondents reported consuming milk and milk products in general. About 16% prepare their own cheeses at home, most of them from pasteurized milk (14.2%) and another 1.7% from non-pasteurized milk (Table 3 questions 3,4), and 16.1% and 24.8% purchase milk and cheese respectively from non-regulated sources (Table 3 questions 5 and 6).

**Table 3:** Prevalence of respondents reporting behaviors associated with **purchase** and consumption of milk products by town with and without reported cases of brucellosis. Percent and Number

	Description of behavior	Total	Type of town with or without reported cases	
		% (N)	With	Without
1	Do you or members of your family drink milk?	96.4 (294)	95.6 (175)	97.5 (119)
2.	Do you or members of your family eat chesses?	91.2 (279)	89.6 (164)	93.5 (115)
3.	At home, do you prepare white cheese from non-pasteurized milk?	1.7 (5)	1.1 (2)	2.5 (3)
4.	At home, do you prepare cheese from pasteurized milk?	14.2 (43)	13.2 (24)	15.8 (19)
5.	Do you purchase milk non-regulated sources?#	16.1 (49)	10.4 (19)	24.4 (30)
6.	Do you purchase white cheese non-regulated sources?	24.8 (76)	26.8 (49)	22.0 (27)
7.	Do you or members of your family eat cheeses that do not come in sealed packaging from non-regulated sources?	40.9 (114)	40.9 (47)	40.9 (67)
8.	Do you eat white cheese that was purchased from non-regulated sources	28.8 (87)	29.8 (54)	27.3 (33)
9.	Do you drink milk that was not purchased in a closed container (not pasteurized)	7.7 (23)	6.0 (11)	9.8 (12)
10.	When you consume food that includes milk products do you inquire if they are made of pasteurized milk?	21.6 (66)	21.4 (39)	22.0 (27)
11.	Many people buy cheeses in the west bank, do you?	2.2 (6)	1.7 (2)	2.4 (4)
12.	Do you purchase cheese from the west bank?	1.3 (4)	1.1 (2)	1.6 (2)
13.	Do you eat cheese when you visit the west bank?	3.9 (12)	4.4 (8)	3.3 (4)

#*p* < 0.001, chi square

When we asked about the consumptions of dairy products, large variations in responses between the questions were given depending on the exact wording. Comparatively a large group of people (40.9%) reported that their family members or themselves consume cheeses that do not come in regulated sealed packaging, suggesting it may be prepared from non-pasteurized milk (Table 3 question 7). However, in another question, asking if the respondent him/herself consumed cheese not from regulated sources, 28.8% responded positively (Table 3 question 8). This may be due to the more specific related question and not wanting to personally admit to a risky behavior. Fewer respondents reported consuming milk, not cheese, that was not pasteurized (7.7%).

Only between 2 and 4% of respondents reported purchasing or consuming milk products from the West Bank, however this may be an under-reporting of the behavior (Table 3 questions 11–13).

In 12 out of the 13 questions regarding consumption of dairy products there was no significant difference between the towns with reported cases of the disease and those without reported cases. In the towns with no reported cases more respondents reported purchasing milk from non-regulated sources compared to towns with reported cases of the disease (24.4% compared to 10.4% respectively, *p* < 0.001) (Table 3 question 5).

Table 4 presents the association between the four major risk behaviors, (purchasing milk or cheese from non-regulated sources and consuming these cheeses) knowledge and attitudes. For all four risk behaviors, attitudes were more favorable towards factors enhancing transmission among those purchasing or consuming these products. Knowledge was lower for those respondents reporting purchasing and consuming milk and cheese from non-regulated sources, however the association was statistically significant only for purchasing milk and personally consuming white cheese.

**Table 4:** The association between attitudes, knowledge and reported behaviors regarding purchase and consumption of milk products

		Knowledge^a^		Attitudes^b^	
Behavior		Mean	Standard Deviation	Mean	Standard Deviation
Do you purchase milk from non-regulated sources?	Yes	3.17	1.43	3.57	0.81
	No	3.73	1.31	2.98	0.96
	P	0.024		< 0.0001	
Do you purchase white cheese from non-regulated sources?	Yes	3.55	1.29	3.52	0.87
	No	3.67	1.36	2.93	0.94
	P	0.30		< 0.0001	
Do you or members of your family eat cheeses that do not come in sealed packaging non-regulated sources?	Yes	3.55	1.32	3.44	0.89
	No	3.70	1.37	2.83	0.87
	P	0.071		< 0.0001	
Do you eat white cheese that was purchased non-regulated sources?	Yes	3.42	1.38	3.53	0.89
	No	3.77	1.28	2.89	0.93
	P	0.015		< 0.0001	

^a^ Mann-Whitney test ^b^T-test

In multivariable logistic regression models the association between the four behaviors (the dependent variables) and attitudes, knowledge and type of town was tested after adjusting for age, gender, education, and income (Table 5). Attitudes were significantly associated with the four risk behaviors presented, indicating that respondents with more favorable attitudes towards factors enhancing transmission of brucellosis also reported purchasing and consuming milk and cheese from non-regulated sources. Knowledge was not associated with any of the four behaviors. Purchasing and consuming cheese from non-regulated sources was more prevalent in towns with reported cases of the disease. However, less milk from non-authorized sources was purchased in towns with reported cases of the disease (even though with borderline statistical significance *p* = 0.075). Goodness of fit was assessed using the Hosmer and Lemeshow statistic, showing no significant difference between the model and observed data (p between 0.25 and 0.73), confirming a good fit of the models to the data.

**Table 5:** Variables predicting purchase and consumption of non-authorized milk products (Multivariable logistic regression models, odds ratio, confidence interval and *p* value)

Do you purchase milk from a non-regulated source? *N* = 272				
		Odds Ratio	P	Confidence Interval
Age		1.02	0.21	0.99, 1.05
Gender^a^		1.21	0.63	0.56, 2.58
Education		1.00	0.98	0.90, 1.11
Income	High	1	–	–
	Mean	0.40	0.08	0.14, 1.13
	Low	0.59	0.41	0.17, 2.07
Type of Town^b^		0.51	0.075	0.24, 1.07
Attitudes		**2.21**	< 0.0001	1.42, 3.43
Knowledge		0.78	0.72	0.59, 1.02
Hosmer and Lemeshow statistics *p* = 0.69, Nagelkerke R^2^ = 0.170				
Do you purchase white cheese from a non-regulated source? *N* = 273				
Age		**1.03**	0.02	1.00, 1.05
Gender^a^		1.02	0.96	0.52, 1.99
Education		1.06	0.17	0.98, 1.16
Income	High	1	–	–
	Mean	0.51	0.14	0.21, 1.24
	Low	0.40	0.11	0.13, 1.24
Type of Town^b^		**2.36**	0.01	1.18, 4.68
Attitudes		**2.66**	< 0.0001	1.80, 3.94
Knowledge		0.89	0.36	0.69, 1.15
Hosmer and Lemeshow statistics *p* = 0.73, Nagelkerke R^2^ = 0.201				
Do you or members of your family eat cheeses that do not come in non-regulated sealed packaging? *N* = 249				
Age		**1.03**	0.02	1.00, 1.05
Gender^a^		0.79	0.43	0.44, 1.42
Education		**1.09**	0.03	1.01, 1.19
Income	High	1	–	–
	Mean	0.97	0.93	0.44, 2.14
	Low	1.07	0.90	0.39, 2.93
Type of Town^b^		1.25	0.44	0.70, 2.24
Attitudes		**2.47**	< 0.0001	1.74, 3.51
Knowledge		0.93	0.52	0.74, 1.16
Hosmer and Lemeshow statistics *p* = 0.25, Nagelkerke R^2^ 0 = .187				
Do you eat white cheese that was purchased from a non-regulated source? *N* = 283				
Age		**1.02**	0.043	1.00, 1.04
Gender^a^		0.83	0.31	0.39, 1.34
Education		1.07	0.12	0.98, 1.16
Income	High	1	–	–
	Mean	0.61	0.26	0.26, 1.42
	Low	0.48	0.19	0.16, 1.42
Type of Town^b^		**2.04**	0.03	1.08, 3.86
Attitudes		**2.50**	< 0.0001	1.74, 3.61
Knowledge		0.86	0.21	0.67, 1.09
Hosmer and Lemeshow statistics *p* = 0.72, Nagelkerke R^2^ = 0.199				

^a^ Reference group- men

^b^ Reference group- towns with no reported cases (0)

Bold- *p* < 0.05

## Discussion

Cases of brucellosis have been reported in various Arab towns in Israel. These are linked to consumption of raw milk, non-pasteurized homemade cheeses or handling infected animals. These dairy products may be consumed privately, given or sold to family, friends and the local community. In this study we assessed, by self reporting, the rates of these behaviors and try to determine if past reported cases of brucellosis in the community is associated with these behaviors.

A large proportion of the respondents reported purchasing and consuming non-regulated dairy products, this may even be under-reported as on the one hand the respondents were aware of the expected behavior and on the other hand may not be aware of where the dairy products comes from. Undesirable behaviors are usually underreported as shown in previous studies [19]. However, we do not know how much of the milk and cheese consumption reported here is from non-pasteurized milk. When pretesting the questionnaire it was clear that people were not sure if the homemade products they consume were made of non-pasteurized milk. But most importantly it is clear that these products could be a source of the disease.

Knowledge is generally regarded as a prerequisite for behavior, the assumption in the past was that if you “know” something is bad for you, you will not consume it [20]. However, knowledge is not enough to drive behavior [21]. In this study, although levels of knowledge were high and even higher in towns that had previous reported cases, there was no association between levels of knowledge and purchase and consumption of non-regulated dairy products after the adjustment for other variables. This implies that just informing the public of the hazards of not pasteurizing the milk is not enough to decrease risk of brucellosis.

Attitudes held by the population towards factors that can enhance transmission of the disease were positively associated with these risk behaviors, also after adjusting for other variables, in multi-variable regression models. Those with more favorable attitudes towards factors that enhance transmission of the disease purchased and consumed more non-regulated dairy products, increasing the risk of contracting the disease. This again is consistent with the literature that suggests that attitudes are correlated with behaviors and many cognitive models are based on this assumption, such as the Health Belief Model [20, 22]. These attitudes included a few concepts. Fatalistic beliefs have been shown to predict risky behaviors among Arabs, such as not using car restraints [23]. According to fatalistic beliefs there is no point in changing behaviors as they do not effect life or health, these are decided by fate or God. Another concept was trust in the source of the homemade dairy products. This trust may be stronger than fear of the disease, maybe due to collectivism. The third concept includes perception of quality of the dairy products, mainly taste and freshness. These qualities seem to be very important and are believed to be much better in homemade products, therefore respondents will accept the risk of the disease and prefer homemade dairy products. This has been reported also in the USA where farm families reported taste and convenience as the main reason for non-pasteurized milk consumption [1, 24]. In Europe, for this reason famous cheeses are made with unpasteurized milk. However, there the farmers comply with the regulations, whereas in Israel this seems to be a problem [6].

All these concepts add up to form strong and deeply engrained attitudes that are based on culture and tradition. There was no difference in attitudes between towns with or without reported cases, suggesting that encountering the disease did not change attitudes. Changing these attitudes may lead to prevention of reported cases, however, they may be difficult to change without a multi-faceted intervention.

The results suggest that in the towns with reported cases, residents consumed more non-regulated cheese, but less non-regulated milk than in towns with no reported cases. This behavior may explain to some extent the reported cases, as in these towns people consumed more cheeses from non-regulated sources, increasing the risk of infection. It seems that encountering cases of brucellosis in the community did not bring about change to traditions of cheese production and consumption. It may be that people found it easier to give up non-pasteurized milk but not the traditional Middle Eastern cheese.

This study suggests an alternative model for the knowledge, attitudes and practices model)KAP (usually referred to as cognitive theories. We suggest that attitudes serve as a value system that provide self-incentives for the continuation of the traditions of dairy consumption, these traditions are risk factors for the outbreaks of brucellosis, and encountering the disease increases knowledge regarding the disease, however does not change behavior. This is in contrast to the more common model where knowledge influences attitudes and attitudes influence behaviors. However, in mass communication research this is often the case and information given to the community via the media, as when an outbreak of the disease occurs, does not necessarily change behavior [25, 26].

It seems that exposure to the disease has not enhanced healthy behaviors as people living in towns with reported cases of the disease consume more cheeses from non-regulated sources. Attitudes towards factors enhancing transmission of brucellosis were associated with consumption of dairy products from non-regulated sources, but knowledge was not. Giving out more information about the disease may not be an effective strategy for controlling the disease.

There may be two options for public health action. The first being the development, implementation and evaluation of a multi-faceted intervention including the community, the media, the healthcare services and local authorities in order to change the norms of consumption of non-pasteurized dairy products. A second option would be to work with the Ministry of Agriculture and the small farmers to secure cattle, goat and sheep free of disease by vaccination and other means, or regulate pasteurization. These public health interventions are only locally and partially performed today, but could be improved and implemented in a wide range of communities and strategies [6, 17, 27].

A major limitation of this study is in the self-reported purchase and consumption of dairy products, however this may be an under-reporting of the behavior as consumption of non-pasteurized dairy products are known to be a source of the disease, or just due to an unawareness that the cheese is not pasteurized. In addition, there may be other differences between the towns that we are not aware of that may confound the results. Another limitation is in the cross sectional methodology that does not permit understanding the directionality of the association or causality, therefore a longitudinal study may help understand the factors affecting the behavior.

## Conclusions

Knowledge regarding the disease and past exposure to the disease within the community do not prevent most risky behaviors related to brucellosis. Interventions should not focus only on dissemination of information.
